# Strategically Funny: Romantic Motives Affect Humor Style in Relationship Initiation

**DOI:** 10.5964/ejop.v12i3.1105

**Published:** 2016-08-19

**Authors:** Theresa E. DiDonato, Brittany K. Jakubiak

**Affiliations:** a Loyola University Maryland, Baltimore, MD, USA; b Carnegie Mellon University, Pittsburgh, PA, USA; Department of Psychology, University of Western Ontario, London, Canada

**Keywords:** humor, humor styles, attraction, romantic motives, short-term relationship, long-term relationships

## Abstract

Not all humor is the same, yet little is known about the appeal of specific humor styles in romantic initiation. The current experimental study addresses this gap by investigating how romantic motives (short-term or long-term) affect individuals’ anticipated use of, and response to, positive humor and negative humor. Heterosexual participants (n = 224) imagined the pursuit of either a desired short-term or long-term relationship, indicated the extent to which they would produce positive and negative humor, and reported how their own interest would change in response to the imaginary target’s use of positive or negative humor. Results revealed that individuals are strategic in their humor production as a function of relational motives. Individuals produced positive humor in both contexts but limited their use of negative humor when pursuing a long-term relationship. The target’s positive humor increased individuals’ attraction, especially women’s, and although negative humor boosted attraction, it did not boost attraction more for short-term than long-term relationships. Findings extend a trait-indicator model of humor and their implications are discussed in light of other theoretical perspectives.

The pursuit of relationships is a worthwhile venture. An abundance of research shows that individuals in committed relationships experience greater subjective well-being (Kamp Dush & Amato, 2005), report fewer mental health problems (Braithwaite, Delevi, & Fincham, 2010), have better physical health (Kohn & Averett, 2014) and live longer (Kaplan & Kronick, 2006) than their single counterparts. Attracting a suitable partner, however, can present a substantial challenge. While individuals may use an array of behavioral strategies to initiate relationships (Clark, Shaver, & Abrahams, 1999), one approach, humor, may be particularly helpful. Indeed, displaying a sense of humor is recognized as *the* most effective tactic that men or women can use to attract a partner (Buss, 1988). Humor, however, is not a unitary construct (Martin, Puhlik-Doris, Larsen, Gray, & Weir, 2003), and how different types of humor are enacted or perceived in a social interaction may be tied to the players’ specific relationship motives (i.e., interest in a short- or long-term relationship). The current study assesses how experimentally-manipulated relationship motives shape the anticipated production and appreciation of different humor styles during relationship initiation. This research provides a nuanced understanding of how specific humor styles are used to attain specific relationships and contributes to the broader discussion of humor’s function during relationship initiation.

## Humor and Attraction

People strongly desire romantic partners who have a good sense of humor (Boxer, Noonan, & Whelan, 2015; Sprecher & Regan, 2002). This preference appears cross-culturally (Lippa, 2007) and is supported by both laboratory (Bressler & Balshine, 2006; McGee & Shevlin, 2009) and field studies (Guéguen, 2010). Why humor holds such wide appeal has been considered from multiple perspectives. One hypothesis contends that humor is a trait-indicator, a sexually-selected behavior that reliably signals genetic fitness by demonstrating unobservable traits like intelligence and creativity (Howrigan & MacDonald, 2008; Miller, 2000) or signals advantageous parent or co-parent qualities, such as warmth and prosociality (Hall, 2015). Another popular theory is that humor may operate as an interest-indicator that allows individuals to test the reciprocity of romantic interest (Li et al., 2009). Other models suggest humor’s function is to signal shared knowledge and compatibility (the encryption hypothesis; Flamson & Barrett, 2008), or to create a pleasant, shared experience (Hall, 2015).

These proposed accounts for humor’s attractiveness largely draw on evolutionary theory. From an evolutionary perspective, sex-based differences in minimal contributions to bearing and rearing children produce sex-linked differences in mating behaviors and partner preferences (Kenrick, Sadalla, Groth, & Trost, 1990). For example, females’ larger minimal investment in any offspring (e.g., gestation) relative to males (i.e., sperm contribution) may explain why women tend to be choosier than men when selecting a romantic partner: their potential costs are higher. They tend to favor men who have the resources and status that demonstrate they can be effective providers (Buss & Barnes, 1986) and men who exhibit traits indicative of a good partner, companion, and co-parent (e.g., warmth; Li, Bailey, Kenrick, & Linsenmeier, 2002). These selection pressures generate intrasexual competition among men, who must then vie to attract a woman’s attention (Buss, 1988). Using humor as a relationship initiation tactic may be one way men, in particular, attempt to gain a competitive advantage.

Evidence supports the effectiveness of humor use as a heterosexual mating tactic. Women are attracted to men’s humor, specifically prioritizing men’s ability to *produce* humor over their willingness to *appreciate* humor (Bressler, Martin, & Balshine, 2006; Tornquist & Chiappe, 2015). Men tend to prefer the reverse pattern, desiring women who appreciate, rather than produce, humor (Bressler et al., 2006). In line with these preferences, women tend to offer less humor production but engage in more humor receptivity (Bressler et al., 2006) or evaluation than men (Wilbur & Campbell, 2011). Accordingly, when making trade-off decisions for long-term partners, women judge men’s humor production as a necessity and their humor receptivity as a luxury, whereas men make the opposite trade (Hone, Hurwitz, & Lieberman, 2015). These findings reflect the sex-linked differences predicted by a trait-indicator hypothesis: men have the burden of displaying favored genetic and relational traits to elicit attraction, and women have the burden of selection in an ambiguous mating context.

Support for the trait-indicator hypothesis is balanced by evidence showing that both men and women initiate and respond to humor when an initial attraction already exists (Li et al., 2009). This interest-indicator model suggests that humor may have evolved as a low-cost mechanism that enables individuals to discern whether someone reciprocates their romantic interest. Humor initiators gather critical information from their target’s (ideally positive) response (e.g., genuine laughter). A test comparing multiple models, however, found minimal support for the interest-indicator model with evidence instead pointing towards trait-indicator models (Tornquist & Chiappe, 2015).

Despite considerable evidence in their favor (Bressler et al., 2006; Tornquist & Chiappe, 2015) trait-indicator hypotheses for humor’s role in relationship initiation have also received mixed support. These hypotheses propose that humor is an honest signal of less-observable but highly favorable underlying traits, such as warmth (Hall, 2015), compatibility (Flamson & Barrett, 2008), or intelligence (Miller, 2000), but humor is not consistently linked to these traits (e.g., Greengross, Martin, & Miller, 2012). Perhaps humor use, generally, may not be a reliable proxy for these traits; instead, the use of specific styles of humor may convey underlying traits most informatively. In other words, the divergent findings linking humor use and favorable underlying traits in previous research might be explained by a failure to distinguish between types of humor.

Consider, for example, the potential link between humor and warmth. When no specific directions are provided to participants about which type of humor to produce, humor production ability is unrelated to producers’ agreeableness (Greengross et al., 2012; Moran, Rain, Page-Gould, & Mar, 2014). Likewise, individuals who advertise more humor production skill on online dating websites do not reliably offer more warmth (Wilbur & Campbell, 2011). Complicating the picture, an analysis of Facebook profiles showed that individuals who produce more humor (in photos) are more agreeable (Hall, 2015), while other work shows that romantic suitors are perceived as less trustworthy than their non-humorous counterparts (Bressler & Balshine, 2006). A closer look suggests that differentiating among humor styles could be a productive way to explain these inconsistent findings. For example, humor production was restricted to examples of negative humor (e.g., self-disparaging humor; flippant humor) in cases documenting an inverse relation between humor and trustworthiness (Bressler & Balshine, 2006; Senko & Fyffe, 2010) and humor and agreeableness (Greengross & Miller, 2008). Likewise, for targets whose dating profiles used unassuming one-liner jokes, which tap the comic and witty style of positive humor, judgments of humor corresponded with perceived warmth (Wilbur & Campbell, 2011). The limited research that directly compares prospective partners’ production of different humor styles, albeit in vignettes, shows that individuals perceive witty or optimistic humor (i.e., positive humor) as indicative of more warmth than disparaging or sarcastic humor (i.e., negative humor; DiDonato, Bedminster, & Machel, 2013). Differentiating among humor styles is clearly necessary in order to evaluate different theoretical explanations for humor’s function in an attraction context.

## Humor Styles and Mate Selection

Humor can be organized along four primary dimensions: two positive styles (affiliative and self-enhancing) and two negative styles (aggressive and self-defeating; Martin et al., 2003). Of the positive styles, affiliative humor often takes the form of wit, jokes, or amusing banter; individuals who use affiliative humor tend to be more extroverted and open, have higher self-esteem and report better psychological health. The other positive humor, self-enhancing humor, is more intrapersonal, focusing on the humorous side of life. Like affiliative humor, its use predicts well-being, less depression, and less anxiety, but its use is also strongly associated with optimism (Martin et al., 2003) and emotional management (Yip & Martin, 2006). Aggressive humor, a negative humor style, entertains at the expense of others and might involve teasing, ridicule, or sarcasm. Its use predicts hostility, aggression, and an inability to perceive others’ emotions; these outcomes are also associated with self-defeating humor (Martin et al., 2003; Yip & Martin, 2006). Self-defeating humor turns the lens on the self through self-disparaging remarks, making fun of the self, or pointing out one’s own weaknesses for amusement. The use of self-defeating humor predicts depression, anxiety, and a variety of psychiatric and somatic symptoms (Martin et al., 2003), as well as Machiavellianism and psychopathy (Veselka, Schermer, Martin, & Vernon, 2010a). These vastly different correlates underscore the need to consider positive and negative humor styles separately in order to understand humor’s role in relationship initiation.

Evidence suggests that both men and women discriminate between humor styles when evaluating potential long-term partners, reporting more romantic interest in hypothetical prospects who use positive humor over those who use negative humor (DiDonato et al., 2013). This is consistent with a trait-indicator model and a contemporary understanding of committed relationships, which acknowledges that both women *and* men make substantial investments to their relationships (Geary & Flinn, 2001) and are therefore highly selective in this context. That people who produce positive humor might be preferred for long-term relationships suggests that perceivers attribute characteristics prioritized for long-term partners (e.g., warmth) to individuals who produce positive humor. Indeed, warmth inferences accounted for the link between positive humor style and long-term interest in previous research (DiDonato et al., 2013). In the short-term context, however, no evidence suggests either gender distinguishes between humor styles (DiDonato et al., 2013). It may be that humor quality (i.e., funniness) is more important than humor style for short-term relationships because humor quality may signal heritable traits (e.g., intelligence) and genetic benefits that women stand to gain in short-term relationships (Gangestad & Simpson, 2000). Having a partner who displays warmth, such as through positive humor use (DiDonato et al., 2013), is typically a welcomed luxury but not a necessity in short-term encounters (Li & Kenrick, 2006). Positive humor might boost a prospect’s attractiveness for a short-term relationship, but, provided the humor is funny, the use of negative humor might be acceptable as well.

Focusing on humor styles rather than general humor use may be one strategy to explain inconsistent findings linking humor and positive traits, as posited by trait-indicator models. However, an even more subtle and refined understanding of humor use may be necessary. Given the evidence that humor style and relationship interest are linked, it stands to reason that suitors may manipulate the presentation of their own humor style as a function of their desire to secure either a short-term or long-term relationship. Indeed, strategic self-presentation is common in relationship initiation contexts (Ellison, Heino, & Gibbs, 2006) and men, who tend to be the humor producers (Wilbur & Campbell, 2011), may be particularly inclined towards strategic humor production. Although humor quality is considered hard to fake (Miller, 2000), skilled humorists may have little trouble adjusting their humor style to help advance their specific relationship goals. For instance, conveying warmth is an effective mating tactic specifically for long-term relationships (Schmitt & Buss, 1996), and if individuals are sensitive to inferences made from specific humor styles, they may strategically display their warmth through positive humor (and refrain from negative humor styles) when initiating a long-term relationship. In other words, triggering a long-term relationship motive could prompt individuals to shift their humor style use towards positive humor over negative humor. In the short-term context, however, the costs of displaying negative humor appear negligible (DiDonato et al., 2013). Since producing negative humor generates more attraction than no humor (Bressler & Balshine, 2006), but may limit inferences of warmth (DiDonato et al., 2013), it may be an ideal strategy for pursuing a short-term relationship. This is echoed by popular pick-up artists who advise that well-timed insults, reminiscent of aggressive humor, are beneficial during relationship initiation (Strauss, 2005). Success at limiting relationships to short-term encounters requires strategic behaviors (Jonason & Buss, 2012), and displaying negative humor at the outset may be part of this repertoire. Thus, negative humor styles (i.e., aggressive humor and self-deprecating humor) may enable individuals to attract others specifically for short-term relationships.

If individuals (especially men) use specific humor styles strategically during relationship initiation, such a finding would support and also expand the trait-indicator model of humor. It would substantiate the idea that evaluators ascribe traits to humor producers based on their humor style, and add new evidence that humor producers anticipate evaluators’ preferences and strategically shift their humor use based on their relational motives. The tendency to use particular humor styles is traditionally viewed as an individual difference variable (Martin et al., 2003); however, we propose that humor styles may be malleable based on motive, which calls into question humor’s suitability as an accurate trait-indicator.

## The Current Study

Humor is a multi-dimensional construct (Martin et al., 2003) yet the majority of studies on humor use during relationship initiation have failed to discriminate among different styles (e.g., Hall, 2015; Wilbur & Campbell, 2011). This failure has contributed to mixed support for different theoretical explanations for humor’s role in social interactions. The work that does differentiate among humor styles shows that positive humor triggers short-term and long-term interest, with inferences of warmth explaining the long-term attraction and offering support for a trait-indicator model (DiDonato et al., 2013). Consistent with this idea, negative humor may be unsuccessful at eliciting long-term interest as it was when individuals evaluated negative-humor producers in vignettes (DiDonato et al., 2013). To promote clarity among different theoretical explanations for humor, the current study examines how triggering different romantic motives affects anticipated humor use during relationship initiation.

Do people opt to strategically use different humor styles to promote their romantic goals? We predicted that both men and women, but particularly men, would demonstrate a refined skill when crafting their presentation of humor in light of their relationship motives, opting for positive humor in pursuit of a long-term relationship or a short-term relationship, and restricting use of negative humor to the short-term context. These results would support humor use as a strategically-modifiable trait indicator and would call into question whether humor producers are uniformly and accurately ascribed positive traits like warmth, as trait-indicator models tend to suggest. Following from literature underscoring the roles of both humor production and humor appreciation (Bressler et al., 2006) we further examined how the production of positive and negative humor in specific relationship contexts would influence romantic attraction. We expected that women, the evaluators (Wilbur & Campbell, 2011), would be particularly sensitive to underlying differences conveyed by humor style, and would report that positive humor increases their interest for both short- and long-term relationships but that negative humor increases interest primarily in short-term contexts.

## Method

### Participants

Undergraduates at a Mid-Atlantic Jesuit University (*n* = 149) and workers on the online labor market Mechanical Turk (*n* = 175) participated in this online study in exchange for psychology course credit or for a nominal fee ($.20), respectively. The advertised eligibility criteria were participant age (at least 18 years old), relationship status (single; not in a romantic relationship), and native language (English speakers). We limited the sample to individuals who met all stated criteria (*n* = 284). We further restricted our sample to those who correctly answered two simple attention check questions (*n* = 245) and completed the booster writing activity (*n* = 242). Lastly, because the study required imagining a potential opposite-sex partner for a long- or short-term relationship, we included only heterosexual participants, resulting in a final sample of 224 participants (167 female; *M*_age_ = 27.12, *SD* = 12.20). Participants predominantly identified as White/Caucasian (80.4%) or Black/African American (8.0%) and generally reported an expected salary of less than 25K (68.8%) or between 25K and 40K (11.6%). Education varied, with 42.0% of participants reporting that they had completed some college, 28.1% reporting that they had completed an Associate’s or Bachelor’s degree, and 21.4% reporting that they had completed high school.

### Design and Procedure

This study used a 2 (humor style: positive or negative) x 2 (relational motive: short-term or long-term) x 2 (gender: male or female) mixed-factor design to investigate the affect of romantic motives on individuals’ humor production and their response to an attractive others’ humor use. Relational motive and gender were between-subjects factors, and humor style was a within-subjects factor.

### Materials and Measures

#### Relational Motives Manipulation and Booster

To manipulate mating motive, we used Griskevicius and colleagues’ (2006) mating primes. Participants read and imagined a detailed scenario in which they had the opportunity for a long-term (*n* = 117) or short-term romantic relationship (*n* = 107) with an opposite-sex individual. In the original mating primes, participants imagined themselves *having* a short-term or long-term relationship. We modified the scenarios in order to maintain a relationship initiation focus: participants imagined that they had an *opportunity* for a short- or long-term relationship and daydreamed about a desired short-term encounter or long-term relationship. Minor modifications were also made to make the primes applicable to non-students as well as students. All participants were reminded mid-study of their mating prime with a booster modeled after Griskevicius and colleagues’ (2006). For 90 seconds, participants wrote in detail about the characteristics that they would desire in the target.

#### Manipulation Check

All participants reported their interest in a short-term and long-term relationship with the target from 1 (not at all) to 7 (very much). Those primed with a long-term motive reported greater long-term interest in the target (*M* = 6.32, *SD* = 0.87) than participants primed with a short-term motive (*M* = 4.56, *SD* = 1.61), *t*(222) = 10.28, *p* < .001. As further evidence of the primes’ effectiveness, participants primed with a short-term motive reported greater short-term interest in the target (*M* = 4.71, *SD* = 1.78) than participants primed with a long-term motive (*M* = 1.99, *SD* = 1.32), *t*(221) = 13.04, *p* < .001. Following Griskevicius and colleagues (2006), we also asked participants to rate their current feelings of romantic arousal and sexual arousal from 1 (not at all) to 7 (very much). As expected, the long-term prime generated greater romantic arousal (*M* = 5.26, *SD* = 1.35) than the short-term prime (*M* = 4.70, *SD* = 1.68), *t*(222) = 3.24, *p* = .001; however, there was no difference in sexual arousal between the short-term (*M* = 3.93, *SD* = 1.74) and long-term (*M* = 3.60, *SD* = 1.71) prime conditions, *t*(222) = 1.42, *p* = .158.

#### Humor Production

Participants were instructed to think about the person they met in the imagination activity and indicate to what extent (1 = never or almost never; 7 = always or almost always) they would engage in each of 16 humor behaviors in order to secure a relationship with the target consistent with their primed motive (i.e., short- or long-term relationship). These humor behaviors were based on Martin et al.’s (2003) Humor Styles Questionnaire. Responses to the affiliative humor (e.g., “I would think of witty things to say”) and self-enhancing humor (e.g., “I would bring attention to the funny side of any awkward situation”) items were combined to form a positive humor subscale (*M* = 4.69, *SD* = 0.84; α = .80). Self-deprecating (e.g., “I would put myself down to make this person laugh”) and aggressive humor (“I would tease this person about something they do or say”) items were combined for a negative humor subscale (*M* = 3.46, *SD* = 0.97; α = .80).

#### Humor’s Effectiveness

Participants were asked to imagine the target in their short- or long-term relationship scenario making them laugh by doing eight humorous behaviors (two representing each of the four humor styles). Participants then reported how each behavior would impact their short- or long-term romantic interest using a scale from -5 (decreases) to 5 (increases). Affiliative humor (e.g., “This person makes you laugh by making warm and witty comments”) and self-enhancing humor (e.g., “This person makes you laugh by commenting on a quirky truth about life”) items were combined to form a positive humor subscale (*M* = 3.16, *SD* = 1.19; α = .82). Self-deprecating (e.g., “This person makes you laugh by making fun of his/her own behavior”) and aggressive humor (“This person makes you laugh by making an off-color joke about someone he knows”) items were combined for a negative humor subscale (*M* = 1.35, *SD* = 1.54; α = .69).

### Procedure

Participants were directed to the online study hosted by Qualtrics. After providing their informed consent, participants indicated their gender and were randomly assigned to complete the motives manipulation, an exercise in which they imagined their own goal of forming a short-term or long-term romantic relationship with an attractive opposite-sex target. A brief manipulation check followed the prime. To test whether individuals alter their humor style based on their romantic motives, we then asked participants to indicate the extent to which they would produce positive and negative humor to secure a relationship with the target. Next, participants completed a booster to remind them of the short- or long-term romantic motive and then reported on the extent to which their own interest in a relationship would be influenced by their partner’s humor use. Finally, participants completed an attention check (i.e., “Where did you meet the person in the story you read?”), provided relevant demographic information, and were debriefed and compensated.

## Results

Correlations between the primary dependent measures are presented in Table 1.

**Table 1:** Means, Standard Deviations, and Correlations among Measures by Gender

Male participants (*n* = 57)	*M* (*SD*)	1	2	3
Anticipated Humor production				
1. Positive humor	4.73 (0.80)			
2. Negative humor	3.58 (1.00)	.54**		
Humor’s effectiveness				
3. Positive humor	2.81 (1.35)	.26	-.05	
4. Negative humor	1.43 (1.45)	.29**	.41**	.44**
Female participants (*n* = 167)	*M* (*SD*)	1	2	3
Anticipated humor production				
1. Positive humor	4.68 (0.86)			
2. Negative humor	3.42 (0.95)	.50**		
Humor’s effectiveness				
3. Positive humor	3.29 (1.11)	.46**	.09	
4. Negative humor	1.32 (1.57)	.44**	.52**	.43**

*p < .05. *p* < .01.

### Anticipated Humor Production

**Figure 1:**
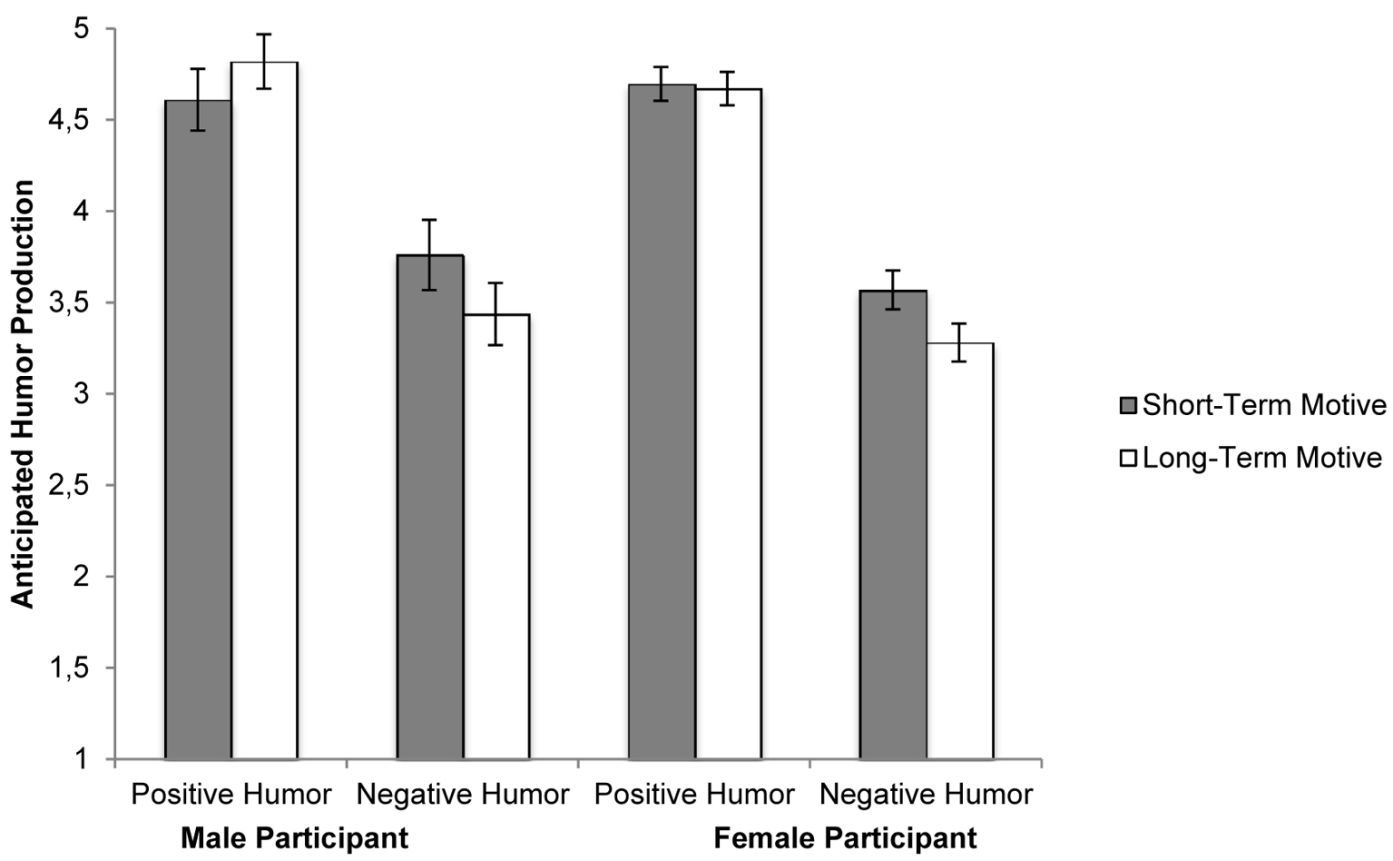
The anticipated production of positive and negative humor as a function of primed relational motive and gender. *Note.* Error bars are 1 *SE* above and below the mean.

To test whether romantic motives shift individuals’ anticipated humor production towards positive or negative humor styles, we conducted a 2 (humor style: positive or negative) x 2 (romantic motive: short-term or long-term) x 2 (gender: male or female) mixed ANOVA (see Figure 1). We observed a main effect of humor style on anticipated humor production: participants rated that they would use more positive humor (*M* = 4.69, *SD* = 0.84) than negative humor (*M* = 3.46, *SD* = 0.97), *F*(1, 220) = 299.72, *p* < .001, η^2^ = .58. We expected that this main effect would be qualified by an interaction with romantic motive. Specifically, we predicted that individuals pursuing both short- and long-term relationships would use positive humor, but that individuals pursuing short-term relationships would use more negative humor than those pursuing long-term relationships. Supporting this prediction, we observed an interaction between humor style and romantic motive on anticipated humor production, *F*(1, 220) = 8.37, *p* = .004, η^2^ = .04. Simple effect comparisons revealed that negative humor was chosen more by individuals pursuing short-term relationships (*M* = 3.61, *SD* = -.93) than long-term relationships (*M* = 3.32, *SD* = 0.99), *F*(1, 220) = 4.23, *p* = .041, η^2^ = .02. There was no difference in the preferred use of positive humor for individuals pursuing short-term (*M* = 4.67, *SD* = 0.91) and long-term relationships (*M* = 4.71, *SD* = 0.78), *F*(1, 220) = 0.49, *p* = .483. Contrary to expectations, participant gender did not independently predict anticipated humor use, nor did it interact with humor style or romantic motive to predict intended humor production (all *F’s* < 1.10).

### Humor’s Effectiveness

**Figure 2:**
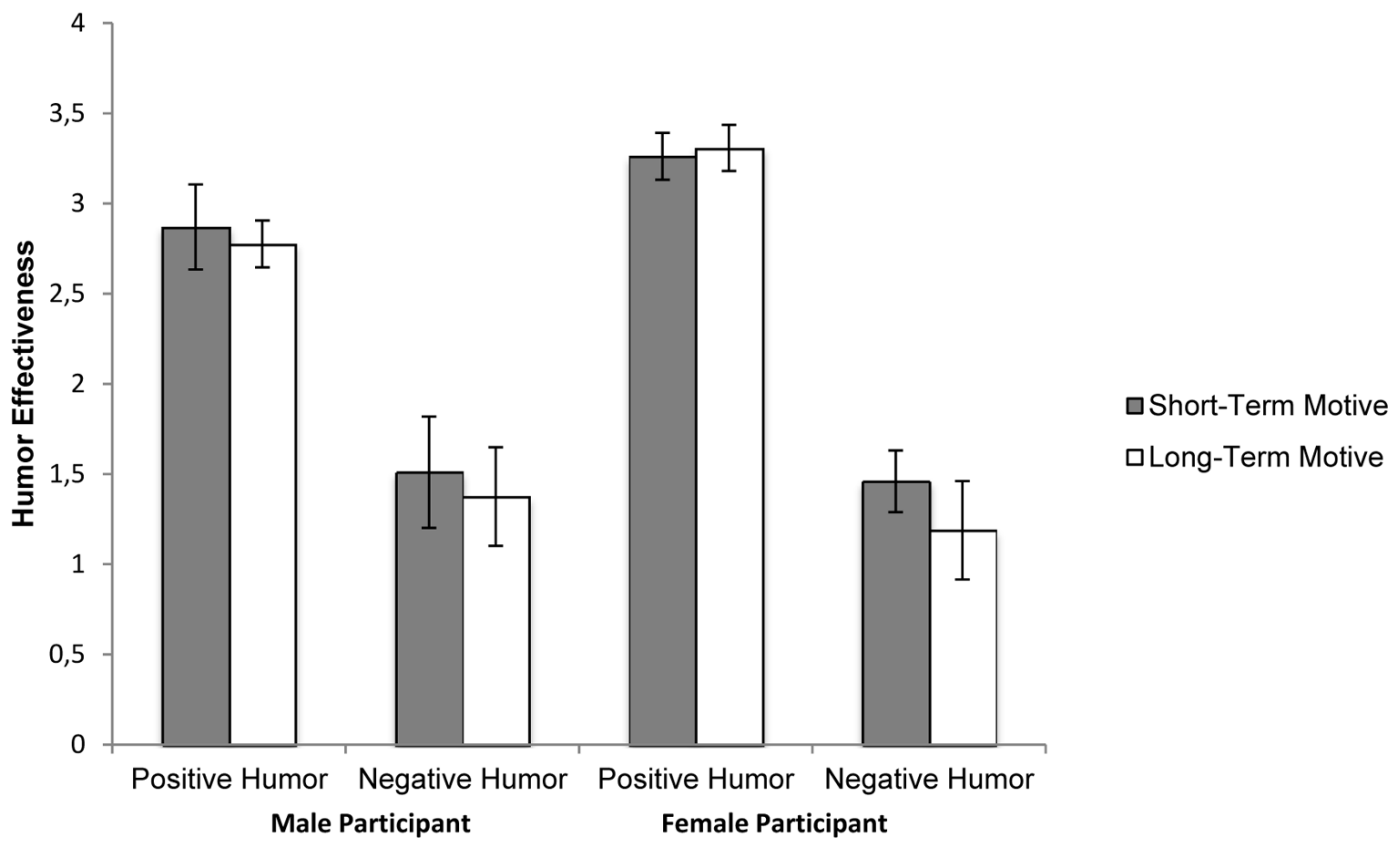
Changes in romantic interest (humor’s effectiveness) as a function of humor style, relational motive, and participant gender. *Note. *Error bars are 1 *SE* above and below the mean.

We predicted that interest in the target would change as a function of the humor style used by the target. Specifically, we anticipated that a target’s positive humor, relative to negative humor, would increase interest for those primed with a long-term motive and for those primed with the short-term motive. We expected the use of negative humor to increase interest more for those primed with the short-term motive than a long-term motive. For this analysis, we used the same mixed ANOVA used to test humor production (see Figure 2).

As anticipated, the target’s use of positive humor (*M* = 3.17, *SD* = 1.19) increased participants’ interest more than negative humor (*M* = 1.35; *SD* = 1.54), *F*(1, 220) = 212.67, *p* < .001, η^2^ = .49. Note that negative humor was still judged to increase attraction, *t*(223) = 71.34, *p* < .001, relative to the mid-point (no change) of the participants’ response scale. This was evident for individuals primed to imagine long-term, *t*(116) = 8.27, *p* < .001, or a short-term relationship, *t*(106) = 10.50, *p* < .001. Contrary to predictions, the observed main effect of humor style on interest was not qualified by motive, *F*(1, 220) = 0.61, *p* = .434. Humor style, however, did interact with gender to predict interest, *F*(1, 220) = 6.41, *p* = .012, η^2^ = .03. Positive humor increased women’s interest (*M* = 3.29, *SD* = 1.11) more than men’s (*M* = 2.82, *SD* = 1.35), *F*(1, 220) = 6.53, *p* = .012, η^2^ = .03. No gender difference (*M*_women_ = 1.32, *SD* = 1.58, *M*_men_ = 1.43, *SD* = 1.45) was observed in responses to the target’s negative humor, *F*(1, 220) = 0.25, *p* = .621. No other effects were observed (*F*s < 1.00).

## Discussion

Scholars have generally examined humor during relationship initiation without making distinctions between different humor styles, despite evidence that humor is a multi-dimensional construct (Martin et al., 2003). Additionally, research on humor styles and trait-indictor theories of humor tend to assume that individuals’ enacted humor styles are fixed and trait-like rather than malleable and motivationally-driven (Martin et al., 2003; Miller, 2000). Perhaps as a result of these limitations, evidence has yet to converge on a specific theoretical explanation for humor’s function in relationship initiation and has supported and contradicted a number of leading hypotheses (Hall, 2015; Tornquist & Chiappe, 2015; Wilbur & Campbell, 2011). The current study builds on existing research (DiDonato et al., 2013) to reveal critical distinctions in the roles of positive humor and negative humor in initial, potentially-romantic, social interactions. Our evidence suggests that people are sensitive to subtle differences in humor styles and prefer to produce the styles of humor that strategically help them pursue specific relationship goals. Individuals reported that they would likely generate positive humor to attract someone for a long-term or short-term relationship, but indicated more willingness to use negative humor when attracting someone for a short-term liaison than a long-term partnership. This distinction gives nuanced attention to how humor might shape others’ impressions and affect their romantic interest. On the receiving end, individuals were attuned to differences in humor style, with their interest increasing more in response to positive humor than negative humor regardless of relational motive.

Our results can be interpreted as partial support for a sexual-selection argument that contends that humor during relationship initiation reveals underlying desirable traits, and they extend this trait-indicator model by calling into question the accuracy of impressions based on humor use. Individuals exhibited what appeared to be strategic inclinations towards using specific humor styles based on their relational motives. Although participants did not actually produce these styles of humor, the observed link between participants’ anticipated humor styles and their primed relational motives renders the production of specific humor styles a potentially imprecise gauge of some underlying traits (e.g., warmth). It follows that humor may not be as honest a signal as proposed by trait-indicator models (Miller, 2000). However, even strategic presentation of humor may rely on possessing the underlying trait to some degree. For instance, an individual who lacks warmth completely may not be able to generate affiliative humor or may generate humor that is only weakly affiliative. In other words, the presence of certain underlying traits may be *necessary* for the successful production of specific humor styles during courtship but not *sufficient*; the producer may require the appropriate relational motive as well. Therefore, humor is likely a reliable indicator of underlying personality traits to some degree, with its honesty augmented by the knowledge of a suitor’s motives.

Prior work links positive humor to perceptions of warmth (DiDonato et al., 2013), and displaying warmth is an effective tactic for securing long-term relationships (Schmitt & Buss, 1996); it makes sense then that individuals would try to attract a desirable long-term partner by producing positive humor. Such a strategy would reflect awareness of the selection pressures to be a good partner in courting for a long-term relationship. Within-person links between the use of positive humor styles (over negative styles) and an array of good-partner traits, such as resiliency, emotional awareness, self-control, and well-being (Vernon et al., 2015; Veselka, Schermer, Martin, & Vernon, 2010b) further underscores the possible appeal of displaying positive humor to potential long-term partners.

Interestingly, participants anticipated producing positive humor in pursuit of a short-term affair as well. Perhaps this reflects a competitive tactic: offering warmth, a luxury in sexual encounters (Li & Kenrick, 2006), might give someone an edge over an attractive rival. Alternatively, positive humor, like any high-quality example of humor, may be used in a short-term context to indicate creativity or intelligence (Greengross & Miller, 2011; Howrigan & MacDonald, 2008), traits that are desired regardless of relational motive (Prokosch, Coss, Scheib, & Blozis, 2009).

Unlike positive humor, producing negative humor, such as teasing or self-targeted humor, carries high risk: it could easily be misinterpreted or yield undesired assumptions about underlying traits (Lampert & Ervin-Tripp, 2006). On account of this risk, individuals might avoid using negative humor when pursuing potential long-term partners because of the negative impression it might create. For instance, aggressive humor may indicate low warmth and discourage potential long-term relationship partners, who tend to prioritize warmth (Li et al., 2002). Less stringent selection criteria for warmth in short-term relationships (Li & Kenrick, 2006) make that context a more viable opportunity to produce negative humor. Alternatively, individuals might actively attempt to showcase negative humor when pursuing a short-term fling, as suggested by some previous research. Specifically, less agreeable men tend to have more success securing casual sexual encounters (Urbaniak & Kilmann, 2006), and women, especially at peak fertility, report more short-term relationship attraction to men who enjoy aggressive behaviors compared to those who do not (Giebel, Weierstall, Schauer, & Elbert, 2013). The qualities potentially inferred from individuals who use negative humor (e.g., aggressiveness, hostility; Martin et al., 2003) may make displaying negative humor a strategic method for initiating short term relationships. Men may also use negative humor toward other men to demonstrate dominance and thereby secure short- or long-term romantic partners. Indeed, men primed with a mating (courtship) motive aggressed more than un-primed men when the audience was male, but not when the audience was female (Griskevicius et al., 2009). Similarly, men may use aggressive humor toward other men but avoid producing negative humor in front of potential long-term mates.

For both humor styles, a sexual selection-based argument anticipates that men would opt to use humor to a greater extent than women. Not only do men typically produce more humor (Wilbur & Campbell, 2011) but humor-producing men and humor-appreciating (not producing) women tend to be favored in mate selection, according to previous research (Bressler et al., 2006; Hone et al., 2015). We did not observe a gender differences in preferred use of humor, which is consistent with other research that has not observed a gender difference in humor production (e.g., Hall, 2015). However, we did not measure actual humor production or humor skill. Anticipated use is not a perfect marker of actual use in a relationship initiation context, suggesting the need for a naturalistic study. Additionally, it is possible that no gender differences were observed because of a relatively low proportion of men in our sample.

Our findings showed that participants’ romantic interest increased when they were on the receiving end of humor, particularly positive humor. This finding also counters an interest-indicator model and supports the idea that favorable information, be it “good parent” characteristics (e.g., warmth) or “good genes” (e.g., intelligence), may have been conveyed by positive humor, thus yielding heightened attraction. As anticipated by sexual-selection models, women were the primary evaluators of humor (Wilbur & Campbell, 2011), reporting greater increases in attraction in response to positive humor than men. Of note, negative humor did not decrease, but rather, *increased* romantic interest, for both long-term or short-term relationships. This is consistent with studies that focus on negative humor (without referring to it as such) and report humor’s positive association with romantic desirability (Bressler & Balshine, 2006; Lundy et al., 1998). There seems to be something fundamental about humor that benefits suitors, independent of their humor style. Perhaps humor’s attractiveness rests in its capacity to signal intelligence regardless of humor style (Greengross & Miller, 2011; Howrigan & MacDonald, 2008). Alternatively, as others have suggested, it could be that humor in general communicates shared knowledge and compatibility (Flamson & Barrett, 2008) or that shared humor suggests a future of pleasant and enjoyable experiences (Hall, 2015). These ideas are not necessarily incompatible with trait-indicator models, as humor may serve many functions related to generating attraction. A study that measures inferred qualities and characteristics from humor in general as well as from different humor styles might add clarity to this question.

This study advances our knowledge of humor in part through its experimental methods, which help reveal that specific relational motives influence individuals’ intended humor tactics and exposure to different humor styles affects romantic interest. The use of hypothetical relationship scenarios and self-report allowed for an initial test of our study’s hypotheses, but research using speed-dating paradigms, peer or observer report, or the coding of observed one-on-one interactions would further substantiate the idea that humor styles are strategically adopted to achieve specific aims, especially if they measured actual, as opposed to anticipated, humor use. We restricted our investigation to romantic relationship initiation, but we would expect different humor styles to influence the beginnings of other relationships as well, such as those between friends, work colleagues, or therapists and clients. Impressions form quickly (Uleman, Adil Saribay, & Gonzalez, 2008) and inferences made from a new acquaintance’s use of positive or negative humor, even if faulty, could have a sustained influence on future interactions (Snyder & Swann, 1978). Research exploring the effect of humor style use in the initiation of non-romantic social relationships would add to our understanding.

Collectively, our findings suggest that humor, which is typically viewed as an honest signal (Miller, 2000) may be, at times, a less reliable predictor of underlying relationship qualities than previously thought. Perhaps the romantic advantage lies with those who choose to strategically generate funny examples of all types of humor. Individuals who know how to tailor their humor style production to match the context may experience the most success obtaining whichever type of relationship they wish to pursue.
